# Ciclopirox and Efinaconazole Transungual Permeation, Antifungal Activity, and Proficiency To Induce Resistance in Trichophyton rubrum

**DOI:** 10.1128/AAC.00442-19

**Published:** 2019-09-23

**Authors:** Daniela Monti, Diletta Mazzantini, Silvia Tampucci, Alessandra Vecchione, Francesco Celandroni, Susi Burgalassi, Emilia Ghelardi

**Affiliations:** a Department of Pharmacy, University of Pisa, Pisa, Italy; b Department of Translational Research and New Technologies in Medicine and Surgery, University of Pisa, Pisa, Italy

**Keywords:** *Trichophyton rubrum*, antifungal resistance, bovine hoof membranes, ciclopirox, efinaconazole, transungual permeation

## Abstract

Onychomycosis is a nail fungal infection, mostly caused by dermatophytes. The treatment efficacy is impaired by difficulties of reaching effective drug levels at the site of infection; frequent relapses occur after cessation of antifungal therapy. The aim of the study was to compare two commercial products containing ciclopirox or efinaconazole for antimycotic activity and antifungal drug resistance. A study of permeation and penetration through bovine hoof membranes, as a nail model, was performed to evaluate the antimycotic activity of permeates against clinical isolates of selected fungi, and the frequency of spontaneous *in vitro* Trichophyton rubrum-resistant strains was assessed by broth microdilution assays. The results suggest that ciclopirox creates a depot in the nail, leading to a gradual release of the drug over time with action on both the nail plate and bed. Conversely, efinaconazole, mildly interacting with nail keratin, mainly exerts its antifungal activity in the nail bed. However, in the case of T. rubrum, the antifungal activities of the drugs in the nail plate seem comparable. Finally, efinaconazole showed a potential for induction of resistance in T. rubrum, which may limit its efficacy over time. Ciclopirox did not show any potential to induce resistance in T. rubrum and appears endowed with a more complete activity than efinaconazole in the management of onychomycosis as the nail keratin is a substrate for the growth of fungal cells, and the availability of drug in large concentration just in the nail bed may not be sufficient to guarantee the complete eradication of pathogens.

## Abstract (precis)

Onychomycosis is a nail fungal infection, mostly caused by dermatophytes. The treatment efficacy is impaired by difficulties of reaching effective drug levels at the site of infection; frequent relapses occur after cessation of antifungal therapy. The aim of the study was to compare two commercial products containing ciclopirox or efinaconazole for antimycotic activity and antifungal drug resistance.

## INTRODUCTION

Onychomycosis is a nail fungal infection mostly caused by dermatophytes and sometimes by yeasts or molds, which can affect part or all components of the nail structure. The most common organisms involved, particularly in toenail infections, are dermatophytes (90%), with Trichophyton rubrum as the main causal agent (71%) (1, 2). Yeast infection due to *Candida* spp. (Candida albicans and/or Candida parapsilosis) accounts for 2% of onychomycosis, and 8% of toenail infections are due to nondermatophyte molds, particularly to Scopulariopsis brevicaulis (3–5). Onychomycosis is typically a chronic infection (disease duration is often >5 years) (6). Furthermore, relapses frequently occur after cessation of the antifungal therapy.

A number of antifungal agents have become available for the treatment of fungal nail infections, and some of them are safe and highly effective in the clinical practice. The systemic agents include terbinafine (TRB) and itraconazole (ITC), which inhibit the synthesis of ergosterol. The local antimycotics include amorolfine (AMF) and ciclopirox (CPX). AMF also acts as inhibitor of membrane ergosterol biosynthesis while CPX acts through the chelation of polyvalent metal cations, leading to inhibition of many cellular activities and modification of the fungal plasma membrane.

CPX exhibits a broad spectrum of fungicidal activity against a number of medically important fungi and is commercially available in two different formulations based on water-insoluble polymers or on water-soluble hydroxypropyl chitosan (HPCH), the latter of which confers on CPX an improved efficacy in the management of onychomycosis (7). In the last years, the new antifungal agent efinaconazole (EFI), a triazole drug that inhibits fungal lanosterol 14 α-demethylase involved in the biosynthesis of ergosterol, has been introduced in the market for the treatment of onychomycosis. EFI displays a broad spectrum of *in vitro* activity against dermatophytes, nondermatophyte molds, and yeasts, showing a more potent antimicrobial activity than the currently marketed antifungal agents (8–10).

Even though the common therapeutic strategies for treating onychomycosis are generally considered effective, resistance against antifungal agents can potentially emerge. Resistance to TRB, a first-line drug in the therapy of dermatophyte infections, is currently increasing in *Trichophyton* clinical isolates (11, 12). By comparing the *in vitro* resistance frequencies and the development of resistance in T. rubrum, we previously demonstrated that mutants resistant to ITC, AMF, and TRB can be isolated (13). No mutant resistant to CPX was found, suggesting no propensity of T. rubrum to develop resistance to this drug. In literature, no evidence of EFI resistance emerged from continuous exposure of T. rubrum to EFI both *in vitro* and in a guinea pig onychomycosis model (14).

In this investigation we analyzed the transungual permeation/penetration of CPX and EFI through bovine hoof slices after topical application of commercial products containing 8% CPX or 10% EFI. Bovine hooves are a well-recognized model for the human nail because of their similarity in terms of composition, consisting of the same keratin network with some differences in matrix density (15–20).

In addition, we evaluated the emergence and evolution of resistance against CPX and EFI in T. rubrum, which was chosen as the model organism for clinically relevant dermatophytes.

## RESULTS

### Permeation/penetration of efinaconazole and ciclopirox through bovine hoof membranes.

**FIG 1:**
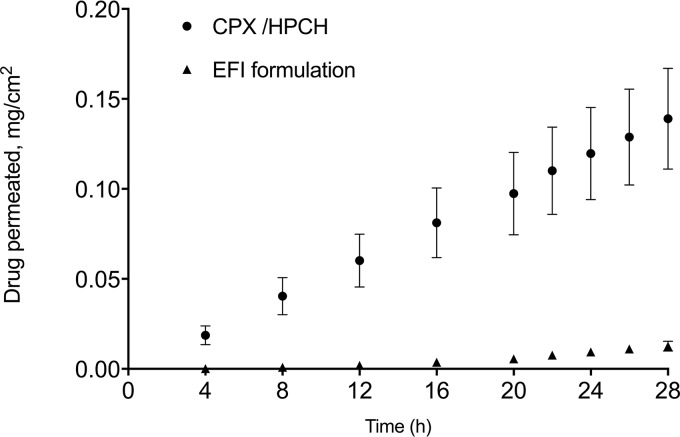
Permeation profiles of ciclopirox (CPX/HPCH) and efinaconazole (EFI formulation) through bovine hoof membranes as a model of human nail permeation.

The permeation studies of CPX/HPCH solution (8% [wt/wt] CPX water-soluble HPCH nail lacquer) and EFI formulation (10% [wt/wt] EFI) were carried out for 30 h. The related permeation profile is shown in Fig. 1. Table 1 summarizes the permeation (flux, *J*; lag time; *P*_app_, apparent permeability coeffcient; *Q*%_30h_, the percentage of permeated drug at 30 h), and penetration (*Q*′_memb_, amount of drug recovered in membrane; *Q*′%_memb_, drug percent [weight/weight] recovered in membrane) parameters for the drugs under investigation.

**TABLE 1:** Permeation/penetration parameters of the drugs through and into bovine hoof membranes

Parameter^*a*^	Value for the parameter^*b*^	
	CPX/HPCH solution (8% CPX)	EFI formulation (10% EFI)
*J* (μg/cm^2^ h)	4.92 ± 0.89	0.58 ± 0.09
Lag time (h)	1.54 ± 0.83	11.03 ± 0.97
*P*_app_ (cm/h) (10^3^)	61.46 ± 11.08	5.82 ± 0.97
*Q*%_30h_	3.29 ± 0.67	0.24 ± 0.05
*Q*′_memb_ (μg drug/mg membrane)	3.37 ± 0.39	0.063 ± 0.01
*Q*′%_memb_	1.32 ± 0.24	0.02 ± 0.005

a *J*, steady-state flux; *P*_app_, apparent permeability coefficient; *Q*, amount of permeant diffusing across the area.

b Values were determined after application of 75 μl of the tested formulations at 30 h (means ± standard errors; *n* = 6). Values for CPX/HPCH solution were significantly different from those for the EFI formulation (*P *< 0.05).

Both tested drugs permeated the bovine hoof membranes, but differences in permeation parameters were noticed related to the active and the formulation. Despite the greater drug concentration in the vehicle (10%, wt/wt), the EFI formulation produced a lower flux of drug (0.58 ± 0.09 μg/cm**^2^** h) accompanied by a long lag time (11.03 ± 0.97 h). The amount of EFI permeated through the substrate at the end of experiments (30 h) after application of EFI formulation was 0.24% ± 0.05% (wt/wt), 5.6-fold lower than that obtained with CPX/HPCH solution (3.29% ± 0.67%, wt/wt). The *P*_app_ value, parameter influenced by the drug chemicophysical characteristics and the type of vehicle independently from the concentration, indicated that the permeability for CPX/HPCH solution was about 10 times greater than that for the EFI formulation. In all cases, the differences between the permeation/penetration parameters of the two products are statistically significant. The CPX/HPCH solution formulation seems to give a higher recovery of drug than the EFI formulation under the same experimental conditions (3.37 ± 0.39 versus 0.063 ± 0.01 μg drug/mg membrane).

Finally, the differences between the two formulations in terms of vehicle type and removal at the end of the permeation tests were analyzed. First of all, we compared a nail lacquer (CPX/HPCH solution consisting of ethylacetate, ethanol, cetostearyl alcohol, hydroxypropylchitosan, and water) and a non-lacquer-based formulation (EFI solution consisting of alcohol, citric acid, C_12_-C_15_ alkyl lactate, cyclomethicone, diisopropil adipate, disodium edetate, and water), the latter chosen to facilitate low surface tension to increase drug uptake. Washing of the membrane at the end of the permeation experiment showed that the EFI formulation was completely removed from the ungual surface exclusively and easily with alcohol, unlike the CPX/HPCH solution, which was easily removed with water.

### Drug concentration and antifungal activity of transungual permeates.

Subungual fluids were collected at different times (4, 8, 12, 16, 20, 24, and 28 h) after application of the nail lacquer CPX/HPCH solution and EFI formulation. Table 2 reports the CPX and EFI concentrations in subungual fluids from the *in vitro* permeation experiments through the bovine hoof membranes as determined by high-performance liquid chromatography (HPLC) analysis.

**TABLE 2:** Ciclopirox and efinaconazole subungual fluid concentrations in the experimental samples

Sample	Time (h)	CPX concn (mg/liter)	EFI concn (mg/liter)
A	4	2.8	1.6
B	8	5.2	1.1
C	12	5.5	1.1
D	16	5.4	1.2
E	20	5.0	1.3
F	24	3.3	1.0
G	28	3.0	0.9

The transungual samples collected at all time intervals were also analyzed for their antifungal activity against three clinical C. parapsilosis, T. rubrum, and S. brevicaulis isolates. As shown in Table 3, antifungal activity against the tested strains was demonstrated for all the samples collected after application of the CPX/HPCH solution and EFI formulation. An inhibitory activity of subungual fluids was measurable from 4 h after administration and was still present after 28 h. Comparable activity of the CPX/HPCH and EFI formulations was obtained against T. rubrum at all time points analyzed since differences of ±1 two-fold dilution are considered not significant in microdilution assays. Statistical differences were obtained for C. parapsilosis at 4, 20, and 24 h and for S. brevicaulis at 16, 20, 24, and 28 h as the subungual fluids obtained after application of CPX/HPCH were less active than those obtained with the EFI formulation.

**TABLE 3:** Minimal inhibitory volume of subungual fluid obtained after application of CPX/HPCH solution and EFI formulation against three fungal strains for each species

Collection time (h)	Minimal inhibitory vol (μl)^*a*^					
	C. parapsilosis		T. rubrum		S. brevicaulis	
	CPX	EFI	CPX	EFI	CPX	EFI
4	100^*b*^	25	6.25, 6.25, 12.5	6.25, 6.25, 12.5	25, 25, 50	12.5, 12.5, 25
8	100	50	6.25, 12.5, 12.5	6.25, 12.5, 12.5	25, 50, 100	25, 25, 50
12	100	50	12.5, 25, 25	6.25, 12.5, 12.5	25, 50, 100	25, 25, 50
16	100	50	12.5, 25, 50	6.25, 12.5, 12.5	50, 50, 100	12.5, 25, 50
20	100^*b*^	12.5, 12.5, 25	25, 25, 50	6.25, 12.5, 12.5	100	12.5, 25, 50
24	100^*b*^	12.5, 25, 25	25, 50, 50	6.25, 12.5, 25	100	25, 50, 50
28	100	25, 25, 50	50	6.25, 12.5, 25	100	25, 50, 50

a In the case all strains in a species are inhibited by the same amount of subungual fluid, only one value is reported. Mode values of six different experiments are given. CPX, CPX/HPCH solution; EFI, EFI formulation.

b Significant difference from the MIC value.

The MIC values of CPX against the selected fungal strains were 0.312 mg/liter for all T. rubrum strains, 0.625 or 1.25 mg/liter for C. parapsilosis, and 1.25 mg/ liter for all S. brevicaulis isolates. The MIC values for EFI were 0.0025 or 0.005 mg/liter for T. rubrum, 0.078 or 0.156 mg/liter for C. parapsilosis, and 0.156 mg/liter for S. brevicaulis isolates.

The effectiveness factor (EF) was defined as the ratio between the expected drug concentration at the site of action and the MIC against each tested strain: the higher the EF, the better the *in vivo* treatment efficacy that could be expected, as reported by Mertin and Lippold (21). Although this observation is true up to a certain level, this is an oversimplification useful only for comparison in the context of the same kinds of experiments. Indeed, beyond a certain ratio an additional increase in efficacy cannot be expected, and moreover efficacy ratios may vary from compound to compound and species to species and cannot be directly compared. In our study two indexes are reported: EF1 refers to the ratio of drug retained in the bovine hoof membranes/MIC of each strain, and EF2 refers to the ratio of drug permeated through the bovine hoof membranes arriving in the subungual space/MIC of each strain.

**FIG 2:**
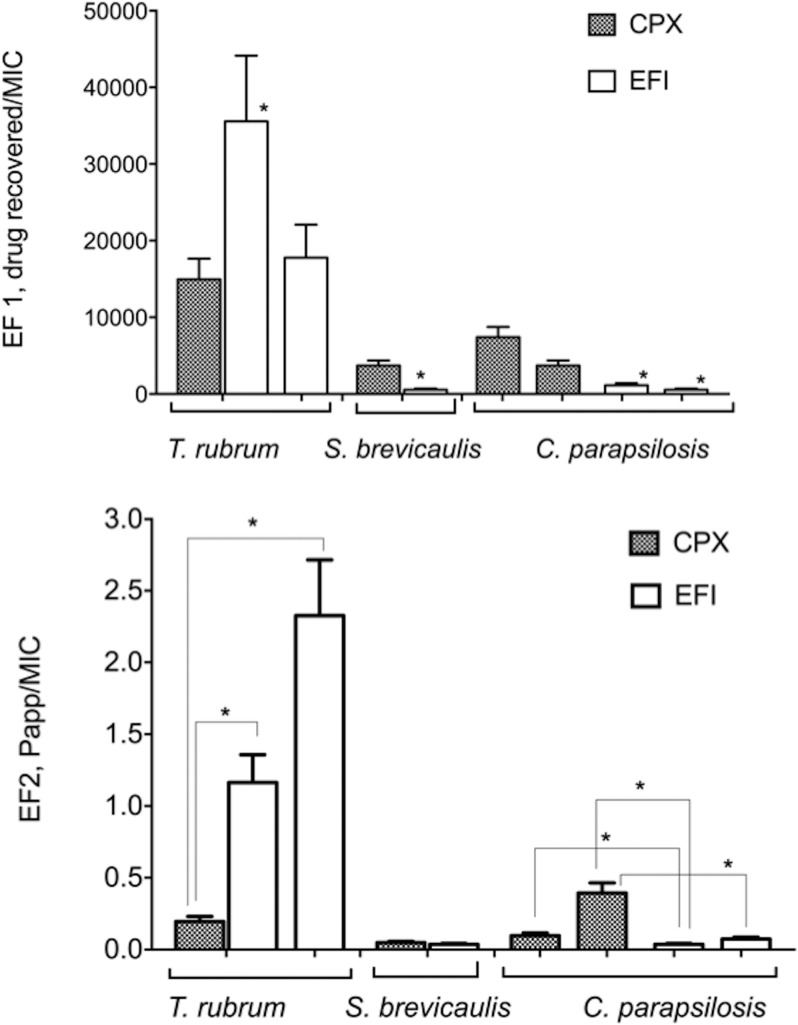
Effectiveness factors (EF1 and EF2) related to ciclopirox (CPX) and efinaconazole (EFI) from CPX/HPCH solution and EFI formulation, respectively. Values are means ± standard errors of the means. *, statistically significant differences. (Top) EF1, the ratio of the drug retained in the membrane at the end of the permeation experiment to the MIC. (Bottom) EF2, ratio of apparent permeability coefficient (*P*_app_) to the MIC.

Figure 2 shows the EF1 and EF2 values calculated for the CPX/HPCH (8% CPX) and EFI (10% EFI) formulations toward the selected fungal strains considering, as the site of action, the drug recovered in (nail plate) and the drug permeated through (nail bed) the membrane, respectively. EF1 values demonstrate that the EFI formulation is more active against T. rubrum when the MIC value of EFI against the strain is 0.0025 mg/liter (*P < *0.01), while the CPX/HPCH solution is more active than the EFI formulation against all S. brevicaulis and C. parapsilosis strains with statistically significant differences (*P < *0.05). In the case of the site of action represented by the drug that permeated in the receiving phase (EF2), CPX appeared more active than EFI against C. parapsilosis (*P < *0.05), to have the same effect on S. brevicaulis, and to be less active against T. rubrum (*P < *0.01).

### Frequency of spontaneous mutants and evolution of resistance to EFI and CPX in T. rubrum.

To evaluate the frequency of spontaneous T. rubrum-resistant strains to EFI and CPX, the T. rubrum strains ATCC 28188, ATCC MYA-4438, CI-1, and CI-2 were grown on Sabouraud dextrose agar (SDA) plates containing the minimal inhibitory drug concentration determined by the agar diffusion assay for each antifungal agent. The frequencies of naturally occurring EFI-resistant mutants for the four strains ranged from 3.3 × 10^−9^ to 2.0 × 10^−8^ (Table 4). All colonies grown on the plates containing EFI were able to grow when transferred to fresh plates containing 1- or 2-fold the amount of drug used for selection. No mutant resistant to CPX was obtained from any of the four T. rubrum strains.

**TABLE 4:** Spontaneous drug-resistant T. rubrum mutants obtained following direct selection on plates containing inhibitory drug concentrations

Strain and drug	Total no. of CFU plated	No. of resistant colonies	Resistance frequency^*a*^
ATCC 28188			
Efinaconazole	2.25 × 10^8^	8	3.5 × 10^-8^
Ciclopirox	4.2 × 10^8^	0	<2.4 × 10^-9^
ATCC MYA-4438			
Efinaconazole	1.6 × 10^8^	4	2.5 × 10^-8^
Ciclopirox	1.6 × 10^8^	0	<6.2 × 10^-9^
CI-1			
Efinaconazole	3.0 × 10^8^	1	3.3 × 10^-9^
Ciclopirox	3.0 × 10^8^	0	<3.3 × 10^-9^
CI-2			
Efinaconazole	4.5 × 10^8^	9	2.0 × 10^-8^
Ciclopirox	4.5 × 10^8^	0	<2.2 × 10^-9^

a Resistance frequency data were calculated by dividing the number of CFU grown on the plates containing each drug by the total number of CFU spread on plates.

To analyze the *in vitro* evolution of resistance to EFI and CPX, the T. rubrum strains (1.0 × 10^5^ CFU/plate) were subcultured (10 transfers) on SDA plates containing 0.5-fold the minimum drug concentration inhibiting growth on solid medium. Abundant fungal growth was obtained with all of the strains using these subinhibitory drug concentrations. After the 5th and 10th transfers, conidia were collected and seeded on SDA plates containing 2-fold the MIC of each drug.

After the 5th transfer, mutants with increased resistance to EFI were isolated from ATCC 28188, ATCC MYA-4438, and CI-2 (Table 5). After the 10th transfer, resistant mutants to EFI were isolated from all tested T. rubrum strains. An increased resistance frequency was observed from the 5th to the 10th transfer. No CPX-resistant mutant emerged following exposure to subinhibitory CPX concentrations after 5 and 10 transfers.

**TABLE 5:** *In vitro* evolution of drug resistance in T. rubrum following exposure to subinhibitory drug concentrations for 5 or 10 transfers before selection

Strain and no. of transfers	Resistance frequency^*a*^	
	Efinaconazole	Ciclopirox
ATCC 28188		
5	7.21 × 10^-8^	<5.24 × 10^-9^
10	6.92 × 10^-7^	<6.75 × 10^-10^
ATCC MYA-4438		
5	1.7 × 10^-8^	<2 × 10^-9^
10	1.25 × 10^-7^	<1.62 × 10^-9^
CI-1		
5	<9.8 × 10^-8^	<4.4 × 10^-9^
10	1.2 × 10^-8^	<2.75 × 10^-9^
CI-2		
5	9.37 × 10^-9^	<5 × 10^-8^
10	2.18 × 10^-7^	<1.19 × 10^-8^

a Resistance frequency data were calculated by dividing the number of CFU grown on the plates containing each drug by the total number of CFU spread on plates.

To evaluate the spectrum of resistance of the isolated mutants, MIC values of EFI, CPX, ITC, and AMF were determined by the broth microdilution assay. We analyzed randomly selected naturally occurring EFI-resistant mutants (spontaneous [S] mutants: 4 strains for ATCC 28188, 2 for MYA-4438, 1 for CI-1, and 5 for CI-2) and mutants obtained after exposure to subinhibitory drug concentrations (induced [I] mutants: 7 strains for ATCC 28188, 6 for MYA-4438, 4 for CI-1, and 7 for CI-2). The parental T. rubrum strains ATCC 28188, ATCC MYA-4438, CI-1, and CI-2 were assayed in parallel. For these strains, the MICs of EFI, CPX, ITC, and AMF were 0.0025 mg/liter, 0.312 mg/liter, 0.078 mg/liter, and 0.16 mg/liter, respectively.

Table 6 reports the MIC values of EFI, CPX, ITC, and AMF for the obtained mutants. Mutants are clustered together on the basis of the parental strain from which they derived and the method used for isolation (S or I mutant). EFI-resistant mutants showed a 4- to 16-fold increase in the MIC values of EFI. Interestingly, some of the EFI mutants showed cross-resistance to ITC, with a 4-fold increase of the MIC values also for that drug. No variations in the MICs of CPX and AMF for EFI-resistant mutants were observed. Analysis of the stability of the EFI-resistant mutants following three transfers on nonselective medium revealed that all mutants were genetically stable.

**TABLE 6:** MIC values of randomly selected induced T. rubrum mutants obtained by the microtiter broth dilution methodology

Parent strain and mutant type^*a*^	*n*^*b*^	MIC (mg/liter)^*c*^			
		Efinaconazole	Ciclopirox	Itraconazole	Amorolfine
ATCC 28188					
S	4	0.01	NS	NS	NS
I	5	0.01	NS	NS	NS
	1	0.02	NS	NS	NS
	1	0.039	NS	0.312	NS
MYA					
S	1	0.01	NS	NS	NS
	1	0.039	NS	NS	NS
I	5	0.01	NS	NS	NS
	1	0.01	NS	0.312	NS
CI-1					
S	1	0.01	NS	NS	NS
I	2	0.01	NS	0.312	NS
	1	0.02	NS	0.312	NS
CI-2					
S	5	0.01	NS	NS	NS
I	6	0.01	NS	NS	NS
	1	0.02	NS	0.312	NS

a S, spontaneous resistance; I, induced resistance.

b *n*, number of strains.

c Mode values of six different experiments are shown. MIC differences of ±1 2-fold dilution were considered not significant (NS).

## DISCUSSION

The intrinsic molecular properties of the active ingredient and the ability of the pharmaceutical formulation to entrap or to release the active ingredients to the site of action have a strong influence on the permeation/penetration of the biological membrane. There are some differences in molecular weight and in partition coefficient that influence the affinity toward the hydrophilic ungual substrate: EFI is more lipophilic (log*P* [where *P* is the partition coefficient] = 3.46; molecular weight [MW] = 348.39) than CPX (log*P* = 2.59; MW = 207.27). Moreover, in previous studies, we demonstrated that the presence of HPCH in the formulation significantly improved the permeation of CPX and AMF through nail membranes (22–24).

In the present investigation, CPX/HPCH solution took advantage both of the better affinity of CPX to hydrophylic ungual substrate and the presence of HPCH. Conversely, notwithstanding that EFI has a low affinity with keratin, which allows having drug free for permeation (25), the EFI formulation seems not to favor the release of the active ingredient. In fact, the nail apparent permeability coefficient is about 10 times lower than that of CPX.

Since fungal infection involves both nail plate and nail bed, we determined the effectiveness of the formulations under study in relation to the amount of drug recovered both in and passed through the bovine hoof membrane. The EF1 of CPX was similar to that of EFI when the MIC of EFI against T. rubrum was 0.005 mg/liter, and it was higher than that of EFI for C. parapsilosis and S. brevicaulis. In the case of the permeation experiments, the EF2 index of EFI was higher than that of CPX for T. rubrum, similar to that of CPX for S. brevicaulis, and lower than that of CPX for C. parapsilosis. In the case of T. rubrum, the higher *in vitro* intrinsic potency of EFI than that of CPX overcame the gap of the lower permeation of EFI in the commercial formulation than that of CPX/HPCH solution. These results suggest that the two drugs act in two different ways: CPX accumulates in the nail, giving rise to a depot leading to a gradual release of the drug over time with action both on the nail plate and in the nail bed. This hypothesis is confirmed by the fact that the washout of CPX from the nail plate was not yet complete 4 weeks after the treatment end, according to a study of Baran and Mailland (26). Conversely, EFI, mildly interacting with the ungual keratin, mainly exerts its antifungal activity in the nail bed; however, in the case of T. rubrum, the two drugs have similar EF1 values, suggesting that their antifungal activities in the nail plate are comparable. In this respect, CPX appears to be theoretically endowed with a more complete activity than EFI in the management of onychomycosis, either superficial or subungual, as the nail keratin is a substrate for the growth of the fungal cells, and availability of drug in large concentration just in the nail bed may not be sufficient to guarantee the complete eradication of the pathogens.

Concerning the last part of our investigation, our results showed a potential for induction of resistance in T. rubrum by EFI, which is not surprising as this drug shares a mechanism of action with other imidazole derivatives, which are fungistatic and induce resistance in dermatophytes (13). Interestingly, EFI-resistant mutants showed cross-resistance to ITC but not to AMF and CPX. In our previous investigation (13), cross-resistance was demonstrated among TRB, ITC, and AMF. Only CPX did not increase its MICs for mutant strains to the other three antifungals, all membrane ergosterol biosynthesis inhibitors.

The potential of EFI to induce resistance in T. rubrum may limit its efficacy over time. In contrast, CPX, which is fungicidal and sporicidal, does not show any potential to induce resistance, and the MICs of the fungi in the spectrum of CPX did not change over several decades of availability of this drug in the clinical practice (27).

## MATERIALS AND METHODS

### Antifungal agents and commercial formulations.

Stock solutions of EFI (MedChem Express, Monmouth Junction NJ), ITC (Sigma-Aldrich, Switzerland), AMF (Sigma-Aldrich, Switzerland), and CPX (Erregierre, Italy) were obtained by dissolving the drug powder (1 mg/ml) in 100% dimethyl sulfoxide (DMSO). The commercial formulations Fulcare (8% [wt/wt] CPX water-soluble HPCH nail lacquer, batch no. 13805B [Menarini Korea]) and Jublia (10% [wt/wt] EFI topical solution, batch no. 9391100 [Valeant Pharmaceuticals, USA]), were obtained from Polichem SA and were used as received. Fulcare has been marketed in other countries with the registered trademarks Ciclopoli, Onytek, Ony-Tec, Onytec, Kitonail, Polinail, Niogermox, Niogermos, Myconail, Rejuvenail, and Privex.

### *In vitro* permeation/penetration experiments.

**(i) Experimental procedure.** *In vitro* permeation experiments were carried out on bovine hoof slices (thickness, 124.4 ± 6.29 μm), employing vertical Gummer-type permeation cells (22, 28). Briefly, 75.0 μl of each commercial formulation was introduced inside the donor chamber, regularly distributed on the membrane surface involved by the permeation (1.40 cm^2^), and treated with warm air for at least 5 min to ensure the removal of the volatile components and the formation of a homogeneous film on the membrane surface. The receiving medium consisted of 5.0 ml of isotonic phosphate-buffered saline (PBS; 66.7 mM, pH 7.4) containing 0.003% (wt/vol) sodium azide to prevent bacterial growth and was maintained at 37°C and stirred at 600 rpm. Each experiment lasted 30 h; at predetermined time intervals, about 5 ml of the receiving medium was collected for analysis and immediately replaced by an equal volume of fresh buffer. Each experiment was replicated at least six times.

To determine CPX and EFI amounts retained by the membranes at the end of the permeation experiments (30 h), bovine hoof slices were carefully cleaned, both mechanically and with water and ethanol, and then weighed. Each membrane was reduced to small fragments, treated with 250 μl of 0.1 N NaOH, and maintained at room temperature under stirring for 24 h; a complete degradation of the keratin structure was obtained. Then 100 μl of methanol was added to the samples to solubilize the drug, and after centrifugation (12,000 rpm, 5 min) a known aliquot was withdrawn, evaporated under vacuum, and reconstituted with a known volume of ethanol to be analyzed by HPLC.

For validation of the extraction procedure, a different series of untreated hoof slices was submitted to the assay, and the retention times of endogenous compounds were compared with those of the drugs under study in order to verify that there was no interference in analyzing the molecules. Moreover, an aliquot of each drug was added to blank membrane, and the extraction recovery was determined by computing the ratio of the amount of drug extracted from the membrane to the amount added. The pecentage of recovery was in the range of 88 to 96%.

**(ii) Analytical methods.** The quantitative determination of drugs under study in the biological samples (receiving phase and bovine membranes) was carried out by HPLC. The apparatus consisted of a Shimadzu (Kyoto, Japan) LC-20AD system with an UV SPD-10A detector equipped with autosampler SIL-10AD VP and a computer integrating system (C-R4A). The injection valve was a Rheodyne with a capacity of 20 μl.

For CPX, the analytical method was previously tuned by using a Phenomenex Synergi Fusion-RP LC column (particle size, 4 μm; pore size, 80 Å; 150 by 4.6 mm), thermostated at 60°C with an HPLC column temperature controller (Thermasphere; Phenomenex). The experimental chromatographic conditions were the following: λ, 220 nm; mobile phase, acetonitrile–20 mM H_3_PO_4_–methanol (50:30:20); flux, 1.0 ml/min; retention time (tr), 4 min.

A Phenomenex Kinetex column (100 by 4.6 mm; pore size, 100 Å; particle size, 5 μm) with a flux of 1.0 ml/min was used for EFI. Isocratic elution was performed using a mobile phase of acetonitrile-water (50:50). The run time was 10 min. A wavelength of 220 nm was used. The retention time was 6.28 min in PBS and 7.14 min in bovine membranes. EFI calibration curve was linear (*r*^2^ = 0.999) in the range of 0.126 to 8.28 mg/liter in in PBS and 0.345 to 4.275 mg/liter (*r*^2^ = 0.994) in hoof membranes. The concentration of permeant in each sample (PBS and biological samples) was determined from standard curves obtained by plotting the concentration of known solutions versus the corresponding peak areas of HPLC chromatograms.

**(iii) Permeation data analysis.** Linear regression analysis of pseudo steady-state diffusion plots allowed calculation of the following parameters: steady-state flux (*J*), given by *Q*/*At*, where *Q* is the amount of permeant diffusing across the area *A* in time *t*; lag time, indicating the time needed by the drug to saturate the membrane and to reach the receiving phase, calculated from the *x* axis intercept values of the regression lines; apparent permeability coefficient (*P*_app_) defined by expression *P*_app_ = *J*/C_v_ where C_v_ is the initial concentration of the drug; percentage of drug permeated at end of the experiment (*Q*%_30h_). Furthermore, the amount of drug (*Q*′_memb_, in micrograms/milligram of bovine hoof membrane) and drug percent (*Q*′%_memb_) recovered in the membranes at the end of the experiment (30 h) were calculated.

All data are the average of six determinations ± standard errors (SE). Statistical differences between permeation parameters were assessed by GraphPad Prism software (GraphPad Software, Inc., San Diego, CA). The evaluation included calculation of means and standard errors, and group comparisons were determined using a Student’s two-tailed unpaired *t* test. Differences were considered statistically significant at a *P *value of *< *0.05.

### Broth microdilution antifungal susceptibility assay.

Broth microdilution assays were performed in accordance with the guidelines of the Clinical and Laboratory Standards Institute (CLSI) in standard M27-A4 for yeast and standard M38-A2 for filamentous fungi (29, 30). The medium used for broth microdilution susceptibility testing was RPMI 1640 medium (Gibco, Gran Island, NY, USA) containing l-glutamine and buffered at pH 7.0 with morpholinepropanesulfonic acid (MOPS). T. rubrum ATCC MYA-4438 was included as a quality control strain for broth dilution antifungal susceptibility testing, as recommended by the CLSI (29).

Stock solutions of antifungal agents were serially diluted using RPMI 1640 medium to yield the concentrations required by the experiments. Drug stocks were serially diluted in RPMI 1640 medium to yield twice the final strength required for the test and added (100 μl) to each well of 96-well microtiter plates (Corning, New York, USA). Yeast were grown on Sabouraud dextrose agar (SDA; bioMeriéux, Marcy l’Etoile, France) for 48 h at 30°C and collected with sterile saline solution. The homogeneous yeast suspension was adjusted to 0.5 McFarland using a Densimat photometer (bioMeriéux, Marcy l’Etoile, France) and subsequently diluted in RPMI 1640 medium to a concentration of 0.5 × 10^3^ to 2.5 × 10^3^ CFU/ml. Stock inoculum suspensions of T. rubrum and S. brevicaulis were prepared from 2-week- and 5-day-old fungal cultures in SDA, respectively. All tested strains well sporulated after this period. Suspensions were obtained from each strain by covering the fungal colonies with sterile saline solution and gently rubbing the colonies with the tip of a transfer pipette. The resulting conidial suspensions were transferred to sterile tubes. Collected conidia were allowed to settle for 10 to 15 min, counted using a hemocytometer, and then adjusted to 1 × 10^3^ to 3 × 10^3^ conidia/ml in RPMI 1640 medium. Plate counts were performed to verify the conidium concentrations by plating aliquots of the adjusted conidial suspensions on SDA.

Inocula (100 μl) were combined 1:1 with the test drug solutions in the microtiter plates. Growth (RPMI 1640 medium–1% DMSO) and sterility (RPMI 1640 medium) controls were included for each tested isolate. The inoculated plates were incubated at 35°C for 48 h or for yeast, up to 7 days for T. rubrum, and 72 h for S. brevicaulis. Each organism was tested in duplicate, and the experiments were repeated three times on separate days. MICs were determined visually, and the MIC endpoint was defined as the lowest concentration that caused a reduction of ≥50% and ≥80% in fungal growth compared to the growth in the control well (drug-free medium) for yeast and mold, respectively. MIC differences of ±1 2-fold dilution were not considered significant.

### *In vitro* antifungal activity of transungual permeates.

Three clinical strains of C. parapsilosis, T. rubrum, and S. brevicaulis originally obtained at the Microbiology Unit of the Pisa University Hospital were used. Strains were propagated from frozen stocks on SDA plates at 30°C. The *in vitro* antifungal activity of transungual permeates was assessed by the broth microdilution methodology. Serial 2-fold dilutions of the subungual fluids were prepared in RPMI 1640 medium according to CLSI guidelines. Fungal inocula prepared as previously described for the antifungal susceptibility assays were added to the microdilution plates. Each organism was tested in duplicate, and the experiments were independently repeated three times. The antimicrobial activity of transungual fluids was defined as the minimal inhibitory volume of fluid that produced at least ≥50% and ≥80% growth inhibition of yeast and mold, respectively.

Two indexes are reported to compare the activity of both drugs: one, taking into account the drug recovered in the biological membrane, effectiveness factor 1 (EF1), was calculated as a ratio of the amount of antifungal agent retained in the bovine hoof membrane per unit of weight to the MIC (micrograms/cubic centimeter/MIC) of the drug against the tested strains, considering the area and thickness of the biological membranes involved; the other, taking into account the drug permeated through the bovine hoof membrane, effectiveness factor 2 (EF2), was obtained as a ratio of the apparent permeability coefficient (*P*_app_) to the MIC of the drug against the tested strains.

### T. rubrum spontaneous resistance and evolution of resistance to antifungals.

Two reference strains (ATCC 28188 and ATCC MYA-4438) and two clinical isolates (CI-1 and CI-2) of T. rubrum were used (13). Strains were routinely propagated on SDA plates at 35°C for 7 days. Stock drug solutions were serially diluted from 0.5 to 0.001 mg/ml in RPMI 1640 medium.

To set up the experimental conditions to test both the natural and the induced resistance to drugs, MIC determinations were performed at inocula of 10^8^ CFU/plate and 10^5^ CFU/plate by the agar dilution method as described by Ghelardi et al. (13). Briefly, serially diluted drug solutions (0.1 ml) were transferred into sterile petri dishes. Liquefied SDA medium (9.9 ml) was added at 45°C and immediately mixed. Control plates were prepared without addition of drugs. A 100-μl volume of the suspensions of dermatophytes was seeded by spot inoculation onto the control plates and on the medium containing the antifungal agents. Plates were incubated at 35°C for 4 days. The antifungal concentration inhibiting growth was defined as the lowest concentration preventing growth of macroscopically visible colonies on drug-containing plates when visible growth was present on the control plates.

The natural resistance to antifungals was evaluated essentially as previously described (13). In brief, conidial suspensions were prepared at 10^9^ CFU/ml, and an aliquot (100 μl) was seeded on SDA plates containing the antifungal agents at the concentrations inhibiting the growth of T. rubrum strains seeded at 10^8^ CFU/plate. Colonies were counted after incubation at 30°C for 3 weeks. The frequency of natural resistance to antifungals was calculated by dividing the number of CFU grown on the plates containing each drug by the total number of CFU spread on these plates. The colonies grown on the plates containing the antifungal agents were transferred to SDA plates containing 1- and 2-fold levels of the drug concentrations used for selection. Growth was checked after incubation for 1 week at 30°C.

The evolution of the antifungal drug resistance was essentially performed as previously described (13). The T. rubrum strains were serially propagated for 10 transfers (7 days of incubation for each transfer) on SDA plates containing 0.5-fold the drug concentration inhibiting growth of each T. rubrum strain seeded at 1 × 10^5^ CFU/plate.

In each transfer, 100 μl of a suspension containing about 10^6^ conidia/ml was inoculated onto SDA plates containing the antifungals, and plates were incubated at 30°C. After the 5th and the 10th transfers, all conidia were collected and seeded on SDA plates containing 2-fold the MIC of each drug.

To ensure that resistant phenotypes were genetically stable in the absence of the drugs, selected mutants were serially propagated for three transfers (7 days of incubation for each transfer) on nonselective SDA plates. After the third transfer, conidia were collected to determine the MIC by the broth microdilution method, as described above. Each organism was tested in duplicate, and the experiments were repeated three times on separate days.
